# Associations between previous sport and exercise experience and physical literacy elements among physically inactive Danes

**DOI:** 10.1186/s12889-021-11299-2

**Published:** 2021-06-29

**Authors:** Peter Elsborg, Clara Heinze, Paulina S. Melby, Glen Nielsen, Peter Bentsen, Knud Ryom

**Affiliations:** 1 Health Promotion Research, Steno Diabetes Center Copenhagen, Gentofte, Denmark; 2 Center for Clinical Research and Prevention, Copenhagen University Hospital—Bispebjerg and Frederiksberg, Frederiksberg, Denmark; 3 Department of Nutrition, Exercise and Sports, University of Copenhagen, Copenhagen, Denmark; 4 Danish School Sports, Nyborg, Denmark; 5 Department of Geosciences and Natural Resource Management, University of Copenhagen, Frederiksberg, Denmark; 6 Department of Public Health, Aarhus University, Aarhus, Denmark

**Keywords:** Physical literacy, Physical activity, Inactivity, Motivation, Self-confidence, Motor skills, Motor competences

## Abstract

**Background:**

Physical inactivity is recognized as a leading global public health threat. Physical Literacy, a concept describing an individual’s prerequisites to participate in and adhere to physical activities, has been suggested to be a key concept in understanding physical activity in various populations. The aim of this study was to describe the prerequisites for physical activity among inactive adults in terms of their physical literacy and previous experience with sport and exercise and how these are interrelated.

**Methods:**

Sample: 1033 physical inactive Danes. Measures: BREQ-3, ESES, the Levels of knowledge questionnaire and the physical self-confidence scale.

**Results:**

Inactive Danish adult’s physical literacy scores are generally low compared to samples in other studies. Inactive adults with no or little previous experience with sport and exercise had lower levels of competences, self-efficacy and autonomous motivation for exercise and sport than the inactive with more experience. Previous sport and exercise experience was positively associated to the physical and affective domain of PL.

**Conclusion:**

Previous experience with sport and exercise is very important to consider when developing sport and exercise activities for currently inactive adults as individuals with low previous experience have lower competences and autonomous motivation and therefore need lower challenges and other important attention to the motivational climate in order to ensure that the activities are motivating enough to secure continued engagement.

## Highlights

1. Previous participation in sport and exercise is positively associated with the affective and physical elements of physical literacy in adulthood.
2. Inactive adults are a heterogeneous group regarding their prerequisites for engaging in physical activities.
3. Inactive Danish adult’s autonomous motivation for sport and exercise is low in comparison to other adult populations.

## Introduction

Physical inactivity (PI) is recognized by the World Health Organisation’s (WHO) as a leading global public health threat [1]. Recent data show that 27.5% of adults do not meet the WHO recommendations on physical activity (PA) internationally, and this number is 25% in Denmark [2, 3]. Physical inactivity (PI) not only affects health risks such as non-communicable diseases, disease-specific mortality, and all-cause mortality [4, 5], but have also been associated with lower mental well-being and quality of life [6, 7]. Globally PI has been estimated to cost health care systems $ (INT$) 53.8 billion in 2013 and contribute to productivity losses of 13.7 billion due to deaths related to PI [8].

In Denmark, despite a solid focus on decreasing the prevalence of PI from the Danish authorities and other stakeholders during the last decade, there has been no positive change in PI in the period 2006–2016 [9]. Thus, the existing approaches to prevent PI seem to have limited effect. Developing and implementing interventions targeted at inactive adults is challenging because inactivity is not a characteristic that individuals physically gather around. Therefore, only few studies have investigating physical activity behaviour in large representative samples of inactive adults [10]. However, this is an important group to gain knowledge about in order to improve and tailor effective initiatives and programs that can increase PA among inactive [10].

**Fig. 1:**
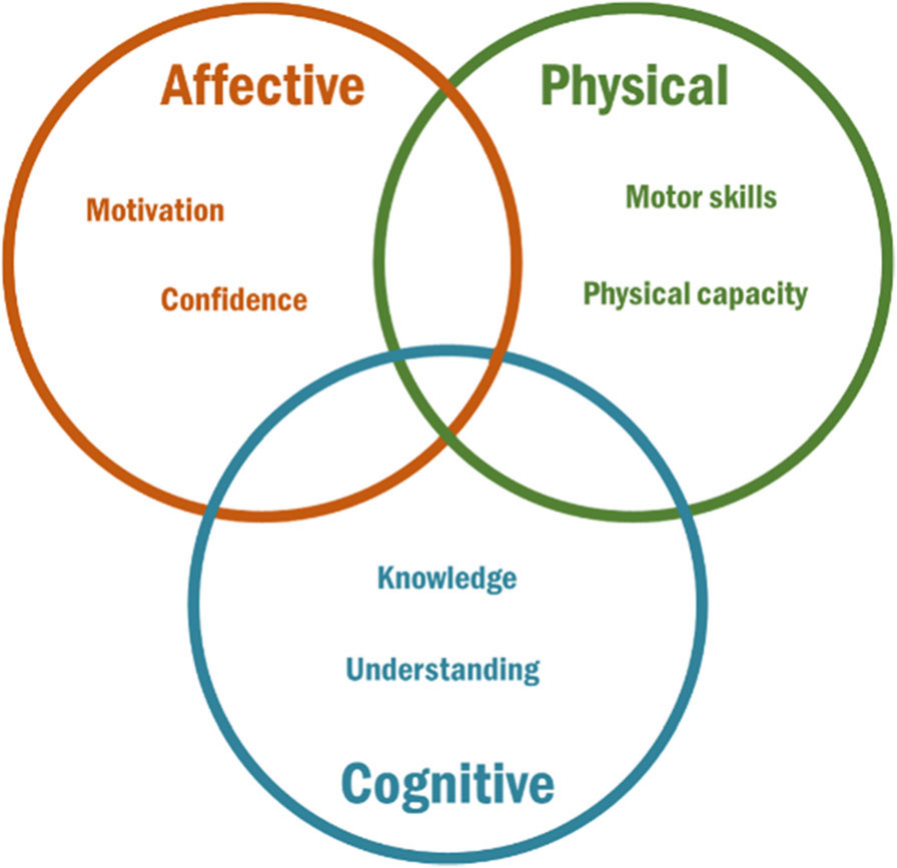
Venn diagram showing how the three domains of Physical Literacy overlap and what concepts they include

The personal resources and prerequisites for PA that different groups of inactive adults have are important for their involvement and engagement in such programs. A concept of potential importance to understanding the underlying causes of PI and potentials for PA is the concept of Physical Literacy [11]. Physical Literacy (PL) is a multidisciplinary and comprehensive concept describing an individual’s prerequisites to participate in and adhere to physical activities throughout the life-course [12]. PL consists of dimensions or elements that lay the foundation for an individual’s capacity and tendency for engaging in physical activities throughout life [13]. Although many slightly different definitions of PL exist, and the domains PL is categorized in has slightly different labels (e.g. the Australian definition has a social domain [14] and one Canadian definition has a behavioural domain [15]), most definitions have elements that can be included in an affective, a physical and a cognitive domain [16]. The affective domain includes elements such as motivation and confidence, the physical domain includes motor skills and physical capacity, whereas the cognitive domain includes knowledge, and understanding (please, see Fig. 1 for an overall model of three broad domains across different PL definitions).

In recent years, the construct of PL has gained increasing attention especially in Australia, Canada and the UK [14, 16, 17]. This recent interest in PL, and its multidimensional nature, stands on a conviction that the combination of these domains brings added explanatory value on PA behaviour than the sum of the individual concepts [13, 18].

The elements within the affective domain are also individual pre-existing scientific areas, within sport and exercise psychology, and the elements within the physical domain, within motor skill research and physiology. All with field-leading experts and with a long record of empirical evidence that shows associations with PA behaviour [19–21]. The scientific history of these domains means that context specific and theory driven measurement tools are developed and validated. However, the cognitive domain of physical literacy is difficult to place as a scientific area on its own, and there has been an ongoing debate within PL research of how to define, operationalize and measure this domain. A pictorial scale has been developed with a holistic view on the cognitive domain of PL [22], however this scale like most of the PL measurement tools developed so far, is developed for children. Most other scales also have the limitation that the cognitive domain is operationalized as knowledge on the health benefits of participating in PA [15, 23, 24]. This leaves many aspects of Whiteheads [11, 12] description and definition about the cognitive domain out. Additionally, research also shows that knowledge about health benefits doesn’t change health behaviour, especially when it comes to health behaviours with a preventive benefit such as PA [25].

PL is developed though participation in different movement activities, sport and exercise activities though life and is therefore highly individual but constantly developed and altered though life in an iterative process as PL influences which activities are possible to engage in meaningfully [11]. It can therefore be hypothesised that adults with different experience with sport and exercise have different levels of PL. This may also be the case for adults who are currently inactive.

The aim of this study was therefore to measure elements of the affective and physical domain of PL in a large sample of Danish inactive adults and analyse how they are related to previous experiences with sport and exercise. This is done in order to inform future sport and exercise programs and initiatives aimed at inactive groups of adults so that they are better suited to different groups of inactive with different prerequisites for participation and engagement in sport and exercise activities.

## Methods

### Procedure

The data collection was conducted by YouGov, which is a global public opinion and data company, in the fall of 2019. YouGov contacted 6196 Danish adults from their user panel. All 6196 were screened for their level of physical activity, using the validated questionnaire IPAQ (International Physical Activity Questionnaire) [26]. One thousand thirty-three of these reported not being physically active at either a moderate or a high intensity during the last 7 days and were therefore invited to answer the full questionnaire (and defined as physically inactive). For the sake of comparison, all 6196 initially contacted respondents answered, a number of background variables such as; gender, age, region, municipality, education, urbanization, marital status, family situation/children in the household, personal income, household income and employment.

### Ethics

The survey was carried out in accordance with relevant guidelines and regulations. All participants were informed that the questionnaire responses were anonymous and voluntary. The Danish Data Protection Agency regulations were adhered to. Institutional ethical approval is not required for anonymous data in social science research in Denmark [27], therefore, no approval was obtained.

### Participants

The 1033 respondents included in our study, were evenly distributed across Denmark. The age category of 18–34-year-old consisted of 24.1% of the participants, 32% were between 35 and 49 years of age and 43.7% were between 50 and 65. Slightly more women than men answered the survey (61% women and 39% men). 31% of the respondents had children at home, most were married (39%), most worked full-time jobs (41%) and the majority have completed a bachelor’s degree or similar (26%). The participants were evenly distributed across Denmark, the largest part (29.8%) were from the capitol region, 13.9% from region Zealand, 22.1% from region southern Denmark, 24% from central region and finally 9.8% from the north region of Denmark. The mean amount of walking per day was 0.46 h (SD = 0.845) while the mean daily sedentary time was 6.25 h (SD = 5.410). This sample of inactive had an average BMI of 28.01 (SD = 6.698).

As panel data has been used in this study, dropout rates are extremely low and representativeness is generally high on demographics, etc., with the single exception that ethnic minorities only make up about 2–4% of YouGov’s user panel, why representativeness is low for this group in this study.

### Measurements

#### Inactivity

IPAQ’s short version (IPAQ-SF), which consists of 7 questions about physical activity within the last 7 days [26], was used to screen for level of physical inactivity, used for inclusion in the study. IPAQ-SF measures moderate and high intensity physical activity in the last 7 days with two items. E.g. “*How many days have you performed hard strenuous physical activities in the last 7 days, e.g. lifting heavy objects, digging, team training in the gym, cycling or running fast?”* IPAQ-SF has been used and endorsed by WHO as a cost-effective method to assess physical activity physical activity.

#### Previous sport and experience

The participant’s previous sport and exercise experience was measured with the questionnaire question; “*which of the following four statements suits you best?”* Followed by a range of four possible answers; “I have never previously participated in sport and exercise”, “I have previously participated in sport and exercise a little”, “I have previously participated in sport and exercise”, “I have previously participated in sport and exercise a lot”. This question was taken from previous large-scale survey in Denmark.

#### The affective domain of physical literacy

To measure the PL elements within the affective domain of physical literacy two previously validated instruments, one for motivation and one for self-confidence was administered.

The behavioural regulation in exercise questionnaire (BREQ-3) was used to measure the participants motivation for exercise. Several studies have provided evidence of the validity of this instrument (e.g. [28, 29]) BREQ-3 measures motivation on the self-determination continuum based on self-determination theory [30]. BREQ-3 consists of 24 items answered on a 5-point Likert scale and measures the six types of motivation of the SDT continuum using 4 items for each motivational score. The questionnaire begins with the overall question “Why do you engage in exercise?”. The six types of motivational regulation are intrinsic regulation (example item: I exercise because it’s fun”), integrated regulation (example item:” I consider exercise part of my identity”), identified regulation (example item: “It’s important to me to exercise regularly”), introjected regulation (example item: “I feel guilty when I don’t exercise”), external regulation (example item: “I exercise because other people say I should”) and amotivation (example item: “I don’t see why I should have to exercise”. Finally, the scales can be combined into a single scale which measures the degree of autonomous regulation. This is called a Relative Autonomy Index (RAI) and is calculated with the following formula: RAI = (intrinsic regulation *3) + (integrated regulation*2) + identified regulation – introjected regulation - (external regulation*2) - (amotivation*3).

The Exercise Self-Efficacy Scale (ESES) was used to measure the participants self-confidence. This instrument has previously been validated in a suitable population [31]. ESES measures one scale that consists of 10 items and is answered on a 4-point Likert scale. The questionnaire begins with the statement “I am confident…”. Example of items are *“that I can overcome barriers and challenges with regard to physical activity and exercise if I try hard enough”*, *“that I can be physically active or exercise even without the support of my family or friends”* and *“that I can be physically active or exercise even when I am tired”*.

Both BREQ-3 and ESES were translated into Danish following the principles of translation back-translation procedure [32]. One researcher translated the items into Danish, another researcher translated the Danish version back to English. The two English versions were compared and where discrepancies were found they were discussed, and the Danish version was edited so that the intention of the original English item came across.

#### The physical domain of physical literacy

For the purpose of this study a questionnaire measuring two elements within the physical domain of PL were developed. These two elements were adult basic movement competencies and ball competencies. The initial idea for the questionnaire was adapted from the Physical Self Perception Profile (PSPP). PSPP measures five separate sub-domains that all examine different aspects of physical self perception [33], and inspired by the validated questionnaire the physical self-confidence scale (PSC), which examines adolescents’ perceived competences for physical activities [34]. As PSC is developed for adolescents, the list of skills was adapted for adults, and focused on basic movement competencies and ball competencies, rather than self-confidence. Prior to this study, the list of questions was pilot tested. One hundred and three participants matching the final sample from the same panel filled in the questionnaire. Response distributions were inspected and the items where response distribution were very skewed were reformulated. Additionally, as a result of the pilot test more explanatory texts was inserted. The final questionnaire consisted of 15 items. The participants were asked to rate a list of skills on a scale from 0 to 10, with 0 indicating that the skill would be impossible to perform and 10 indicating that the they would be able to perform this skill every time without mistakes. Examples of skills within the basic movement competencies scale are:” Balancing on a bench on one leg”, “Sidestepping on a straight line” and “Jumping forward from a standing position”. Examples of skills within the ball competencies scale are “*Kicking a ball placed in front of you on the floor?”, “Dribbling a ball with your hand five times in a row in a standing position” and “Throwing an overhead throw with a tennis ball”.* As previous studies have showed that it is important to consider the possible overlap between motor competence and fitness when trying to measure competence, the participants were deliberately asked whether they were able to perform the task without making mistakes or not and not for how long they were able to do it in order to avoid measuring fitness [35]. ,The items were chosen, so that each scale both included very easy tasks (such as sprinting in a straight line) and more difficult tasks (such as balancing on a bench on one leg).

Because no previously validated measure for the cognitive domain of physical literacy has been developed for adults and because it was not within the resources of this survey to develop one that could capture this sparsely researched domain of PL, it was decided that this domain would not be measured.

### Statistical analysis

The data were analysed using the Statistical Package for the Social Sciences (SPSS) version 25.0. To describe the sample of inactive and the included study variables, table of frequencies (percentages) and descriptive statistics (mean ± standard deviation) were used. As suggested by Kim (2013) [36] that for samples exceeding 300, skewness values either below − 2 or above 2 should be considered problematic.

The measure of the physical domain was developed for the purpose of this study as described earlier in the method section. This meant that the validity of the scales needed to be tested and that the factor structure determined. This were done using Exploratory Factor Analysis. Maximum likelihood estimation was used as the extraction method. For rotation method, an oblimin rotation with Kaiser Normalization was used. To determine a factor structure, the Kaiser’s criterion (eigenvalues ≥1), a scree plot, and the interpretability of obtained factors were carefully considered. Loadings on the intended factor above .3 and below .3 on unintended factors were used as guiding values for item retention. Cronbach alpha was calculated to estimate the internal reliability of the scales. Cronbach alpha values above .7 were considered acceptable.

Pearson R correlation tests were conducted to determine the association between the included variables, with the r-coefficient reported.

Four unadjusted and four adjusted generalized linear models were conducted in order to explore associations between previous sport and exercise experience and the elements of the affective and physical domain of physical literacy, with Estimated Marginal Means (EMM) and 95% CI reported. Adjusted models were adjusted for age, gender, educational status and the three remaining PL elements. The reference category for the previous sport and exercise experience variable was *“I have never previously participated in sport and exercise”*.

## Results

### Factor validity of questionnaire measures of the physical domain

An exploratory factor analysis revealed a two-factor solution to the items measuring basic movement competences and ball competencies (See Table 1). Nine items formed a factor reflecting basic movement competences. All items had high loadings on intended factor (.611–.964). One item (jumping over a small obstacle) had a cross loading slightly above the .3 guiding value (.312). However, as the loading on its intended factor was high and the theoretical interoperability of this being a basic movement skill, it was decided to retain the item in the intended scale. Six items formed a factor reflecting the ball competencies scale. All items in this scale had high loadings on intended factor (.687–903). None of these items had loadings on the unintended factor above .3.

**Table 1:** Loading matrix from an exploratory factor analysis for the questionnaire measuring physical competences

	Basic movement competences	Ball competences
Jumping in a straight line	**0.96**	−0.04
Sprinting in a straight line	**0.92**	−0.12
Jumping three times on your right leg and then on your left	**0.92**	−0.04
Running in a straight line	**0.90**	−0.02
Jumping forward from a standing position	**0.87**	0.09
Jumping up in the air from a standing position	**0.85**	0.09
Sidestepping on a straight line	**0.74**	0.12
Balancing on a bench on one leg	**0.70**	0.12
Jumping over a small obstacle	**0.61**	0.31
Catching a tennis ball with two hands	−0.09	**0.90**
Throwing an overhead throw with a tennis ball	−0.04	**0.89**
Rolling a ball along the floor with underhand grip	0.02	**0.83**
Dribbling a ball with your hand five times in a row in a standing position	0.03	**0.79**
Kicking a ball placed in front of you on the floor	0.20	**0.70**
Hitting a non-moving object in front of you at hip height with a bat	0.18	**0.69**

Loadings on intended factor bolded

### Descriptives, validity and reliability of PL elements

Table 2 displays descriptive statistics of the measured scales in this study. All scales measuring the four PL elements showed acceptable Cronbach’s alpha values (.846–.966) above the threshold of .7 indicating high internal consistency of the scales. No scales from the affective domain or from the physical domain showed problematic skewness values.

**Table 2:** Descriptive statistics of study variables

	Mean	Std. Deviation	Skewness	Cronbach alpha	Minimum	Maximum
**Affective domain**						
Intrinsic regulation	2.889	1.439	0.399	0.913	1	5
Integrated regulation	2.597	1.380	0.809	0.866	1	5
Identified regulation	3.053	1.273	0.491	0.846	1	5
Introjected regulation	2.783	1.433	0.564	0.887	1	5
External regulation	1.984	1.367	1.669	0.900	1	5
Amotivation	2.250	1.453	1.235	0.915	1	5
RAI	3.502	7.642	0.088	–		
Exercise self-efficacy	2.777	0.747	−0.464	0.924	1	4
**Physical domain**						
Physical competence	7.122	3.097	−1.022	0.966	0	10
Ball Competence	7.873	2.575	−1.514	0.934	0	10

*RAI* relative autonomy. index. *N* = 1033

### Correlations between PL elements

Table 3 displays the correlation matrix for autonomous motivation (RAI), exercise self-efficacy, physical competence and ball competence. All PL elements were significantly positively correlated with the one another (*p* < .001). Autonomous motivation showed a moderate correlation to the other element in the affective domain exercise self-efficacy and weak correlations to both the scales of the physical domain. Exercise self-efficacy showed moderate correlations to all the other three PL element scales. Physical and ball competence had high internal correlation.

**Table 3:** Pearson’s Correlations and descriptive results of study variables

Variable	RAI	Exercise self-efficacy	Basic movement competence	Ball competence
Relative Autonomy Index	–			
Exercise self-efficacy	0.354***	–		
Physical competence	0.135***	0.449***	–	
Ball competence	0.173***	0.361***	0.752***	–

* *p* < .05, ** *p* < .01, *** *p* < .001

### Associations between elements of physical literacy and previous sport and exercise experience

Table 4 describes the associations between previous sport and exercise experience and physical literacy elements. As can be seen the inactive adults with no previous experience with sport or exercise had significantly lower levels of autonomous motivation, self-efficacy, and ball- competence than those who had just a little; some or a lot of experience. They also had lower levels of basic movement competences than the groups with some or a lot of experience. The level of autonomous motivation increased gradually with the different amounts of experience with sports and exercise from negative autonomous motivation among the inactive with no previous sport and exercise experience to about 9 times as high level of autonomous motivation among inactive with a lot of previous sports and exercise experience. Adjusting for the demographic background variables gender, age and education did not change the pattern of differences between groups. However, when also adjusting the associations for the other PL elements, previous sport and exercise experience was still related to autonomous motivation and ball competence while, but not to self-efficacy or physical competence.

**Table 4:** Four unadjusted and four adjusted general linear models of previous sport and exercise experience and elements of the affective and physical domain of physical literacy

Experience with sport and exercise	No experience	A little	Some	A lot
**RAI**				
EMM (95% CI)	−2.11 (REF) (−3.44; −0.78)	2.59*** (1.94;3.24)	5.19*** (4.22;6.16)	7.15*** (6.23;8.06)
EMM adjusted^a^ (95% CI)	−2.79 (REF) (−4.38; −1.21)	1.66 *** (0.60;2.72)	4.44 *** (3.19;5.69)	6.33*** (5.15;7.52)
EMM adjusted^b^ (95% CI)	−1.99 (REF) (−3.51; −0.48)	1.86*** (0.85;2.87)	4.17*** (2.97;5.36)	5.71*** (4.58;6.84)
**Self-efficacy**				
EMM (95% CI)	2.52 (REF) (2.39;2.65)	2.70** (2.63;2.76)	2.88*** (2.79;2.98)	3.00*** (2.90;3.09)
EMM adjusted^a^ (95% CI)	2.60 (REF) (2.45;2.76)	2.75 * (2.64;2.85)	2.91*** (2.79;3.03)	3.00*** (2.89;3.12)
EMM adjusted^b^ (95% CI)	2.79 (REF) (2.65;2.94)	2.79 (2.70;2.89)	2.81 (2.70;2.92)	2.87 (2.77;2.98)
**Basic movement competences**				
EMM (95% CI)	6.55 (REF) (6.00;7.09)	7.00 (6.73;7.27)	7.85*** (7.45;8.25)	7.72*** (7.34;8.10)
EMM adjusted^a^ (95% CI)	7.10 (REF) (6.47;7.74)	7.37 (6.94;7.79)	8.10** (7.60;8.60)	7.85* (7.38;8.37)
EMM adjusted^b^ (95% CI)	7.79 (REF) (7.36;8.22)	7.51 (7.23;7.79)	7.65 (7.32;7.99)	7.47 (7.16;7.79)
**Ball competence**				
EMM (95% CI)	7.14 (REF) (6.70;7.58)	7.87*** (7.66;8.09)	8.53*** (8.21;8.85)	8.40*** (8.10;8.71)
EMM adjusted^a^ (95% CI)	7.18 (REF) (6.66;7.71)	7.85 ** (7.50;8.20)	8.49*** (8.08;8.91)	8.36*** (7.97;8.75)
EMM adjusted^b^ (95% CI)	7.41 (REF) (7.04;7.79)	7.84** (7.60;8.09)	8.00*** (7.71;8.29)	7.98*** (7.71;8.25)

* *p* < .05, ** *p* < .01, *** *p* < .001. Reference category (REF): I have never previously participated in sport and exercise. Adjusted models are adjusted for age, gender, educational status and the three remaining PL elements

*EMM* estimated marginal mean. ^a^Models adjusted for the demographic variables age, gender and education. ^b^Models adjusted for the same demographic variables and all other PL elements

## Discussion

This is the first study to investigate the level of PL elements in a large representative sample of inactive adults. The study showed that the level of motivation, and self-efficacy of the affective PL domain and the elements ball competence and motor competence of the physical domain among inactive adults depended on their previous experience with sport and exercise. When adjusting these associations for the interdependence among the PL elements, sport and exercise experience was only significantly associated to the affective element autonomous motivation and the physical element ball competence. Together with the finding that elements within the domains very strongly correlated this indicates that previous sport and exercise experience has independent and separate importance to both the physical domain and the affective domain of physical literacy. These results are in accordance with both the theoretical foundation of PL [12, 13] as well as empirical studies linking exercise participation and PL [37–41], however this study is the first study to show this in a large sample of inactive adults. The two elements in the affective domain; autonomous motivation and self-efficacy were moderately strong correlated, whereas autonomous motivation domain was only weakly correlated to elements of the physical domain. It is in concordance with the theory of PL that elements within each domain are strongest correlated [15, 40]. However, it is also in line with other research that perceived self-efficacy for PA is also related to actual competences for PA [34]. That self-efficacy and perceived competences are related to autonomous motivation is also in line with self-determination theory (SDT) based research which shows that experiences of competence are important for intrinsic motivation [20].

When comparing to other samples of adult populations of about the same mean age, where BREQ has been used to measure motivation for exercise, the inactive adults in our study show lower levels of autonomous motivation (intrinsic, integrated and identified motivation). A sample of north American adults (mean age = 36 years, 84% women) with varying levels of daily PA reported higher levels of autonomous forms of motivation; intrinsic regulation (14%) and identified regulation (22%) [42]. In a Spanish study a sample of adults (mean age = 32 years, 53% women) participating in non-competitive recreational sport and physical activities reported higher levels of intrinsic regulation (64%) and identified regulation (22%) and lower amotivation (− 17%) [43]. A sample of Portuguese adults (mean age = 36 years, 64% women) participating in 5 different modes of exercise and sport had much higher levels of intrinsic motivation (45%), integrated motivation (61%), identified motivation 47%), and lower amotivation (51%) [29]. In a sample of parents to schoolchildren (mean age = 41 years 72% women) higher levels on intrinsic regulation (21%) and identified regulation (19%) and lower. Amotivation was observed (− 44%) [44]. Altogether, it can be concluded that the inactive Danish adults show generally low scores on autonomous motivation. This is not surprising as many studies have shown bi-directional associations between autonomous motivation and physical activity [45]. It is however an important point to be aware of when trying to promote and create physical activity options for this target group as autonomous forms of motivation has been shown to be important for continuation in exercise and sport [45–48]. The use of BREQ which is a a widely used questionnaire to measurese motivation for exericse made it possible to compare with other samples of adults. Unfortunatly, this was not possible to do with regards to the other elements measured in this study, since the measure for the physical domain is newly developed it has not been used in other samples and because the measure for self-efficacy has mainly been used in samples with disease comparisons on this element did not make sense either. Future studies should investigate if healthy inactive adults also have lower scores in both the physical and cognitive domain of PL compared to adults with different activity levels.

### Practical implications

The results of this study have several important practical implications. They point out that the part of the population categorised as physically inactive a prime target group for public health interventions, have quite different back grounds relating to their experience with, competence and motivation for sport and exercise. Inactive adults cannot and should not be viewed as one homogenic group where one solution fits all. It is an important finding that, depending on their previous experience with sports and exercise, physically inactive adults have very different resources for engaging and participating in sport and exercise activities in terms of motivation, self-efficacy and competences [10, 49].

It is especially important to be aware that the group of inactive with no previous experience with sports and exercise have very low autonomous motivation, low self-efficacy and low competences for sport and exercise compared to the groups with more experience. As the experience of flow as well as of competence is important to autonomous and especially intrinsic motivation for and enjoyment of an activity [50] it is important to tailor the challenges met in the activities so that they match these different levels of competence. The results of our study show that merely asking participants about their previous experience with sport and exercise seems an easy way to base such differentiation of challenges and perhaps distribution of participants to different exercise groups and activities with different difficulty level. Another both striking and important finding is that the group of inactive adults with no previous experience with sport and exercise had a negative RAI score for autonomous motivation for sport and exercise. This means that they have no desire, or personal will to be physically active and that the only motivation they feel is external pressure [30]. This is a challenge for recruiting this group for exercise activities and even more so for securing their continued participation once recruited [10]. It must therefore be the prime focus for any activities aimed at this group to increase their autonomous motivation for sport and exercise, as this type of motivation is crucial to sustained continuation [45, 48]. A lot of research has shown that such autonomous motivation is dependent on participants experiencing competence, relatedness and autonomy when doing the activities [20]. The satisfaction of these 3 basic psychological needs in sports and exercise activities are often secured when the group climate and the coach has a high focus on tasks instead of performance [51], when the coach or trainer supports social relations and autonomy instead of being controlling the participants and when there is a good match between the challenge of the activity and the participants competences [52].

Future studies should investigate how these results could be translated into interventions that might enhance inactive adults PL and activity levels. All though PL interventions for this target group at the moment are lacking it must be mentioned that a protocol for an holistic exercise intervention with the aim of increasing inactive adults PL was recently published [49].

### Strengths and limitations

The study has several limitations that should be kept in mind. One limitation of this study as a study of PL is the lack of a measure for the cognitive domain. The cognitive elements knowledge and understanding of physical activity that enable PA engagement is probably an important prerequisite for lifelong activity albeit a difficult one to define and hence measure. It was chosen not to include such a measure because a previously validated instrument was not available, and it was not within the resources of this study to do develop one. Additionally, when PL is viewed as a broad holistic concept, the elements measured in this study does not cover all aspects of PL. Future studies should investigate other elements within PL and its importance for inactivity, and here the 30 elements developed in Australia could serve as inspiration for what elements to investigate [14]. However, for the purpose of this study a questionnaire-based measure intended for measuring the physical domain of PL amongst inactive adults was developed and validated using exploratory factor analysis. Two elements; reflecting fundamental basic movement competencies and more specific ball competences were identified as two different phenomenon (factors). With items loading highly on intended factors and high Cronbach alpha values these measurement scales seem both reliable and valid measures for ball and physical competence amongst inactive adults. However, other studies should confirm this factor structure and the different types of validity of the scales among other more active samples. Although optimally the physical domain of PL should be measured objectively with physical testing, including perceived PL elements are highlighted in a recently published scoping review as an advantage when trying to measure the physical domain of PL. [53]

Another limitation to this study is the use of panel data. Using panel data for data collection comes with pros and cons. It gives fast and easy access, but on the other hand there is a risk of participants just trying to finish as fast as possible in order to recieve the gift cards there are provided with upon completion of a specific number of questionnaires as incentive. However, the panel data provider has software installed in their data collection system, ensuring that respondents can’t choose randomly and not filling in the questionnaire correctly. Our analysis of the data has also ensured us on the quality of data. Additionally, the use of panel data ensured the representativity and the size of the sample in this study which is a strength.

## Conclusion

Inactive adults with no or only little previous experience with sport and exercise have lower levels of competences, self-efficacy and autonomous motivation for exercise and sport than the inactive with more experience. This indicates that participation in sport and exercise is important for development of these elements of physical literacy.

It also indicates that inactive adults cannot and should not be viewed as one homogenic group where one PA promoting solution fits all.

Therefore previous experience with sport and exercise may be very important to consider when developing sport and exercise activities for currently inactive adults as individuals with low previous experience need lower challenges and other important attention to the motivational climate in order to ensure that the activities are motivating enough to secure continued engagement.

## Data Availability

The datasets used and/or analysed during the current study available from the corresponding author on reasonable request.

## Abbreviations

WHO: World Health Organisation’s
PA: physical activity
PI: Physical inactivity
PL: Physical Literacy
IPAQ: International Physical Activity Questionnaire
BMI: Body mass index
BREQ-3: Behavioural Regulation in Exercise Questionnaire version 3
RAI: Relative Autonomy Index
ESES: The Exercise Self-Efficacy Scale
PSPP: Physical Self Perception Profile
PSC: physical self-confidence scale
EMM: Estimated Marginal Means
